# Supplementary material for: Association between oral microbiome and seven types of cancers in East Asian population: a two-sample Mendelian randomization analysis
Source: Front Mol Biosci. 2023 Nov 21;10:1327893. doi: 10.3389/fmolb.2023.1327893 (PMC10702768; doi:10.3389/fmolb.2023.1327893)
Supplement: Supplementary file 1 [file DataSheet1.ZIP › Supplementary Materials/MR plots of saliva/saliva═╝/ovarian cancer/pheno.2746_to_ovarian cancer_leave_one_out.pdf]

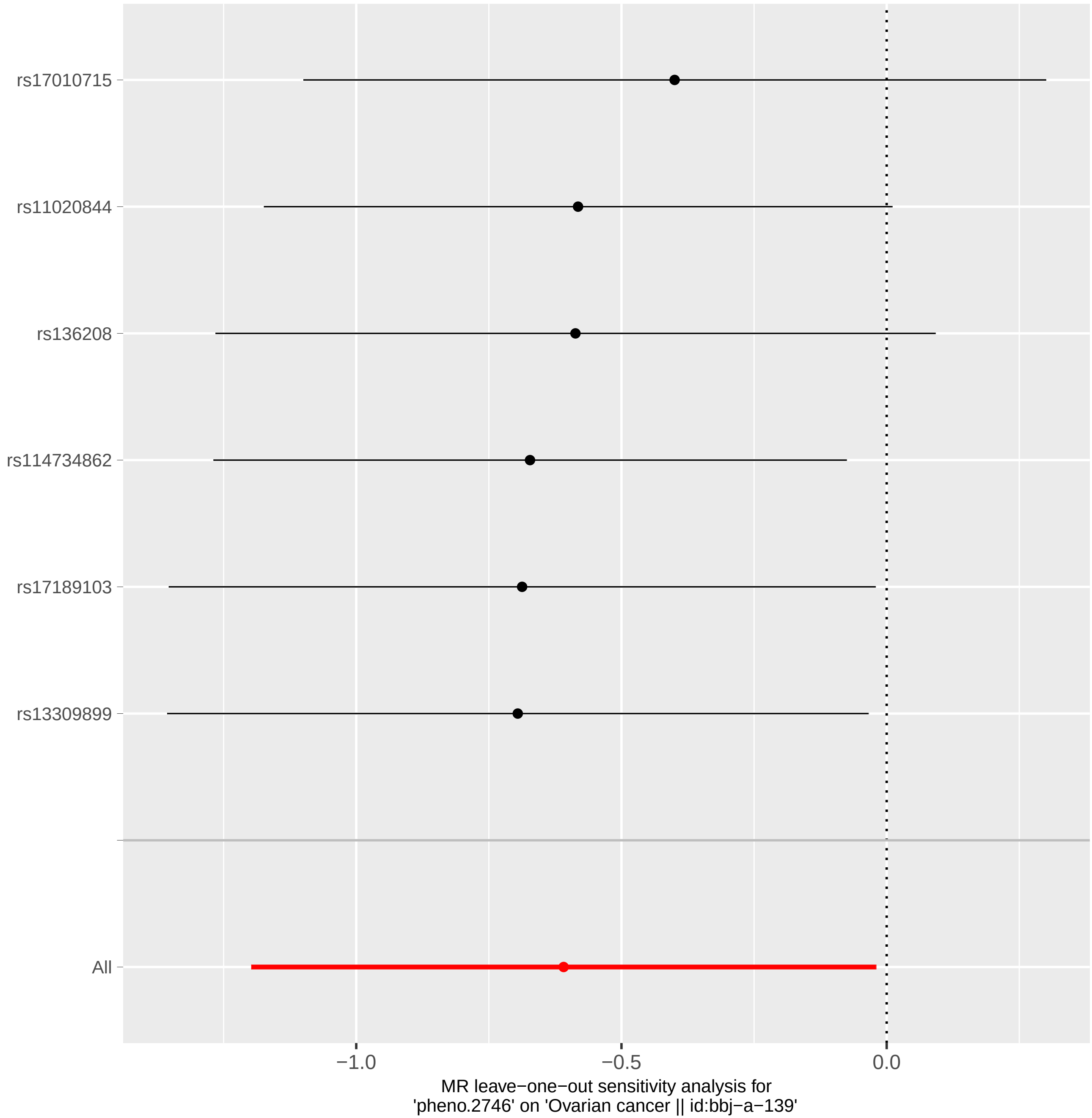
rs17010715
rs11020844
rs136208
rs114734862
rs17189103
rs13309899
All
−1.0
−0.5
0.0
MR leave−one−out sensitivity analysis for
'pheno.2746' on 'Ovarian cancer || id:bbj−a−139'
